# Gout impacts on function and health-related quality of life beyond associated risk factors and medical conditions: results from the KING observational study of the Italian Society for Rheumatology (SIR)

**DOI:** 10.1186/ar4281

**Published:** 2013-09-03

**Authors:** Carlo Alberto Scirè, Maria Manara, Marco Amedeo Cimmino, Marcello Govoni, Fausto Salaffi, Leonardo Punzi, Maria Cristina Monti, Greta Carrara, Carlomaurizio Montecucco, Marco Matucci-Cerinic, Giovanni Minisola

**Affiliations:** 1 Epidemiology Unit, Italian Society for Rheumatology, Via Turati 40, 20121, Milano, Italy; 2 Division of Rheumatology, IRCCS Policlinico San Matteo Foundation, University of Pavia, Viale Golgi 19, 27100, Pavia, Italy; 3 Clinica Reumatologica, Dipartimento di Medicina Interna e Specialità Mediche, University of Genova, V.le Benedetto XV 6, 16132, Genova, Genova, Italy; 4 Rheumatology Unit, Department of Medical Sciences, University of Ferrara Azienda Ospedaliero-Universitaria S. Anna, Via Aldo Moro 8, 44124, Cona, Ferrara, Italy; 5 Department of Molecular and Clinical Sciences - DISCLIMO, Polytechnic University of Marche, Via Tronto 10/a, 60020, Torrette di Ancona, Ancona, Italy; 6 Rheumatology Unit, Department of Medicine DIMED, University of Padova, Via Giustiniani 2, 35122, Padova, Italy; 7 Department of Public Health, Experimental and Forensic medicine, University of Pavia, Via Forlanini 2, 27100, Pavia, Italy; 8 Department of Rheumatology AVC, Department of Biomedicine & Division of Rheumatology AOUC, Department of Medicine & Denothe Centre, University Florence, Largo Brambilla 3, 50134, Firenze, Italy; 9 Rheumatology Unit, Azienda Ospedaliera San Camillo, Circ. Gianicolense 87, 00152 Roma, Italy

## Abstract

**Introduction:**

Gout is the most prevalent arthritis and significantly impacts on function and quality of life. Given that gout associates with disabling comorbid conditions, it is not clear whether such a complex of diseases accounts for the increased disability or if gout may play a role by itself. This study aims to evaluate the specific influence of gout and disease-related features on functional disability and health-related quality of life (HRQoL) in patients with gout followed in rheumatology clinics.

**Methods:**

A random sample of patients was drawn from clinical registries of 30 rheumatology clinics across Italy. Sociodemographic, general health and gout-specific variables were collected. Functional disability and HRQoL were assessed by the health assessment questionnaire (HAQ) and the Physical and Mental Component Summary scores (PCS and MCS) of the Short Form-36 (SF-36). Crude and adjusted ordinal logistic and linear regression models were applied to investigate the specific contribution of different variables on HAQ and SF-36 scores. Results are presented as odds ratio (OR) or mean difference (MD) and 95% confidence intervals.

**Results:**

Out of 446 patients with gout, 90% were males with a mean age of 63.9 years and median disease duration of 3.8 years; the majority of patients were overweight or obese, and with several comorbidities; 21.1% showed at least moderate disability; the PCS score was significantly lower than expected age- and gender-matched samples in the general population, while MCS score was not. After adjusting for potential sociodemographic and general-health confounders, gout-specific variables significantly impacted on HAQ, including polyarticular involvement OR 3.82 (1.63, 8.95), presence of tophi OR 1.92 (1.07, 3.43) and recent attacks OR 2.20 (1.27, 3.81). Consistent results were found for PCS. The impairment of PCS compared to the general population was limited to patients with features of chronic gout. MCS was only affected by recent attacks (MD -2.72 [-4.58, -0.86]) and corticosteroid treatment (-3.39 [-5.30,-1.48]).

**Conclusions:**

The data from the KING study confirm that gout impacts on disability and provide evidence for an independent association of gout and gout-related features with functional outcome and HRQoL. This result supports the need to improve specific treatment in gout.

## Introduction

Gout is the most common arthritis in adults with a worldwide prevalence of 1 to 2% [1]. In Italy the prevalence of gout is lower, ranging from 0.5 to 0.9%, although it is rapidly increasing as in other western countries [2,3]. Population aging, increased drug utilisation and changes in lifestyles and dietary habits may account for this increase over time [4].

In patients with gout, functional disability [5], impairment of health-related quality of life (HRQoL) and increased mortality have been reported [6]. Gout therefore emerges as a major public health issue. However, it is well known that gout is associated with several cardiovascular risk factors including hypertension, dyslipidaemia, insulin resistance, obesity, as well as renal failure [7]. Whether the impact of gout is related to the disease itself or whether it primarily arises from associated risk factors and medical conditions is still unclear [8].

Today, effective treatments are available for gout but it has not yet been determined whether, in order to minimise long-term detrimental outcomes, its prevention and management should primarily target risk factors and comorbidities rather than disease mechanisms [9]. The relevance of assessing patient-oriented outcomes, such as functional ability and HRQoL, has been recently recognised by the Outcome Measures in Rheumatology Clinical Trial (OMERACT) [8,10,11].

Previous case-control and cross-sectional population studies have shown that HRQoL and functional impairment were mainly due to confounders, including sociodemographic characteristics and comorbidities, rather than due to gout itself [12,13]. These studies did not explore clinical characteristics in depth and hence drew conclusions on the overall population of patients with gout without determining the influence of specific features of the disease. Gout has a wide clinical heterogeneity that may reflect the varying impact on physical ability and HRQoL. Prognostic studies on convenience samples of patients with gout investigated the association between disease characteristics and outcomes; such studies identified as predictors acute symptoms, presence of tophi, previous and current joint involvement, and, under specific conditions, urate-lowering treatment (ULT), but they often selected chronic and refractory gout without controlling for several confounders [9,12,14-19]. Whether gout and gout severity variables themselves are independently associated with poor functional ability and HRQoL remains to be confirmed [8].

In 2011 the Italian Society for Rheumatology (SIR) established the Kick-off of the Italian Network for Gout (KING), a national multicentre cohort study that recruited patients with gout selected by random sampling from rheumatology centres across Italy. Using cross-sectional data from this ongoing study, this analysis explored the influence of disease-related features, derived from a detailed clinical examination, on functional ability and HRQoL in patients with a clinical diagnosis of gout and who are followed in rheumatology clinics. Potential confounders including sociodemographic variables and general health variables, such as comorbidities and lifestyle, were taken into account. The results of these analyses allowed one to evaluate the specific impact of gout, across its spectrum of disease severity, on functional disability and HRQoL.

## Patients and methods

### Study design and recruitment

This is a cross-sectional analysis of an ongoing multicentre cohort study including patients with a clinical diagnosis of gout from 30 rheumatology centres in Italy (KING Study, promoted by SIR; NCT01549210) recruited between June 2011 and January 2012. All Italian rheumatology centres were asked to participate. The cohort is a nationwide representative sample of patients referred to rheumatology clinics.

To limit selection bias arising from nonprobability samples (for example, consecutive sampling) resulting from the limited time span of recruitment and infrequent follow-up appointments [20], participants were selected from clinical registries by random cluster sampling. Rheumatologists sent a list of all patients registered in the previous 2 years at their clinics with a diagnosis of gout. Patients were then randomly selected from these registries by an independent investigator (CAS) at a central facility who also checked for duplicates between centres by using probability record linkage based on gender, date and place of birth. The selection complied with the Italian data protection regulations.

Rheumatologists confirmed from their clinical records that the patients selected had a diagnosis of gout. Patients were recruited by telephone following a rigorous contact protocol to optimise recruitment of patients. Patients included in the study sample did not systematically differ from the source population in terms of age, gender and follow-up duration.

A total of 450 patients were recruited but four of them were excluded because at baseline assessment they did not meet the inclusion criterion of clinical diagnosis of gout. The study flowchart is reported in Additional file 1.

The study protocol was approved by the ethics committees of the following institutions: IRCCS Policlinico S. Matteo Foundation of Pavia, San Martino Hospital of Genoa, University Hospital of Bologna, University Hospital of Ferrara, University Hospital of Firenze, University Hospital of Foggia, University Hospital of Messina, University Hospital of Padova, University Hospital of Palermo, University Hospital of Siena, University Hospital of Verona, University Hospital of San Camillo, Forlanini Hospital of Rome, Humanitas Institute of Milano, G. Pini institute of Milano, S. Giovanni Battista Hospital of Torino, Macchi Foundation of Varese, and the local health authorities of Barletta, Catania, Crotone, Cuneo, Marche, Milano, Roma, Pescara and Salerno. The study was conducted in accordance with the Declaration of Helsinki, and each patient provided written informed consent.

### Clinical assessment

At baseline all patients underwent full clinical evaluation, which followed a structured case report form that included both general health and disease-specific variables.

General variables included sociodemographic data, lifestyle factors, the food-frequency questionnaire of the Italian National Institute for Statistics for national surveys, and comorbidities scored according to the Self-Administered Comorbidity Questionnaire [21,22]. The Self-Administered Comorbidity Questionnaire score ranges from 0 to 36 since it includes 12 medical problems and allocates 1 point for the presence of each problem, another point if the patient receives treatment for it, and an additional point if the problem causes a limitation in function [21].

Gout-related variables included symptom duration, classification according to the 1977 preliminary American College of Rheumatology criteria [23], disease-related comorbidities (hypertension, dyslipidaemias, diabetes, cerebrovascular and cardiovascular disease and urinary stones), pattern of disease (such as number and site of involved joints, presence and site of tophi), previous and current treatment for gout, joint examination on 66/68, measurement of tophi, a 0 to 10 numeric rating scale for pain, disease severity, general health and serum uric acid (sUA) concentration.

All patients completed the Italian versions of the Health Assessment Questionnaire Disability Index (HAQ-DI) and the Short-Form-36 (SF-36) [24,25].

To limit information bias and to improve overall reliability, all rheumatologists were instructed to collect the data and clinical measurements by following standard definitions and operating procedures. All data were also centrally checked for missing values and inconsistencies through a standardised procedure of data monitoring and cleaning.

### Variables: outcomes, predictors and confounders

Functional disability assessed by the HAQ-DI was the primary outcome measure of this analysis. The HAQ-DI is a self-administered questionnaire validated for several forms of arthritis including gout and is recommended by OMERACT [10]. The HAQ-DI score (range 0 to 3) was categorised into three different classes of functional disability according to relevant cutoff values proposed by the developers and as applied in other arthritides: absent or mild (0 to 1), moderate (>1 to 2) and severe (>2 to 3) [26,27].

HRQoL measured by the SF-36 questionnaire was the secondary outcome. The SF-36 is a widely used, self-administered general health status instrument consisting of 36 items, which can be scored as two summary scores: Physical Component Summary (PCS) and Mental Component Summary (MCS) scores. The summary scores were normalised to the Italian population, where the mean score is 50 with a standard deviation of 10 [25,28,29]. A score below the mean score of 50 implies a lower health status as measured by the PCS and MCS. Clinically significant difference for the SF-36 summary scores was set at 2.5 as previously reported [30].

Disease-related variables were used as predictors and were coded as follows: symptom duration (>5 years), cumulative joint involvement (1 joint, monoarticular; 2 to 4 joints, oligoarticular; >4 joints, polyarticular), number of attacks in the last year, attacks in the last month, presence of tophi, number of swollen joints, number of tender joints, sUA (<5 mg/dl; ≥5 to 6 mg/dl; ≥6 to 7 mg/dl; >7 mg/dl) [31], current ULT, current nonsteroidal anti-inflammatory drugs or colchicine use, and systemic corticosteroid use at any time (previous or current).

The following general variables were classified as confounders: age, sex (male as reference), education (none or primary; secondary, upper secondary and tertiary education), employment (employed; unemployed; retired; unknown/other), smoking (current smoker; nonsmoker or exsmoker), body mass index category (underweight, ≤18.5; normal weight, 18.5 to 24.9; overweight, 25 to 29.9; obesity, ≥30), high alcohol consumption (>0.5 litres of wine per day, beer more than occasionally or spirits more than occasionally).

Sample size was estimated based on the primary objective of the cohort study; that is, the evaluation of a prognostic model of progression of functional disability after 12 months of follow-up including a training set of 300 patients and a validation set of 150 patients.

### Statistical methods

Descriptive measures of sociodemographic, general health and gout-related variables are presented as the absolute and relative frequency, mean and standard deviation or median and interquartile range based on their type and distribution.

Age-stratified and gender-stratified Italian normative data for the SF-36 were used to calculate the expected HRQoL for a standard population of similar age and gender structure [28]. The relationship between predictors and the HAQ score was modelled using ordinal regression [32]. Proportionality of odds across the HAQ groups was checked for each regression. Results are presented as odds ratios and 95% confidence intervals. The relationship between predictors and secondary outcomes (PCS and MCS) were analysed using linear regression models and the results are presented as mean difference and 95% confidence intervals.

To evaluate the specific effect of gout-related variables, three different set of models were used for each outcome: unadjusted; adjusted for age and gender; and adjusted for age, gender, education, employment, body mass index, smoking, alcohol consumption and comorbidities. To compare expected with observed PCS scores, mean (and 95% confidence interval) PCS scores according to different disease characteristics (disease duration, joint involvement, presence of tophi and number of attacks) and presence of comorbidities were obtained fitting fully adjusted linear regression models.

The overall percentage of missing information was less than 1% for variables included in the analyses. Given the high number of variables (*n *= 21), complete case analysis would have included only 83% of patients. Missing data were imputed using chained equations [33] and the fully adjusted analyses were performed on 10 multiple imputed datasets.

Analyses were performed using STATA software package (2009, release 11; StataCorp, TX, USA).

## Results

A total of 446 patients with a clinical diagnosis of gout were included in this analysis. The main sociodemographic, general health and disease-related variables are summarised in Table 1.

**Table 1:** Patients' characteristics

Characteristic	
Sex (male)	403/446 (90.4)
Age (years)	63.9 (11.6)
Employment	
Employed	137 (30.7)
Unemployed	28 (6.3)
Retired	226 (59.6)
Unknown/not declared	15 (3.4)
Education	
No or primary	158 (35.9)
Secondary	121 (27.5)
Upper secondary	123 (27.9)
Tertiary	38 (8.6)
Current smokers	72/444 (16.2)
Body mass index	28.0 (4.1)
High alcohol consumers	160 (36.4)
Comorbidities	3 (1 to 4)
Hypertension	313 (70.3)
Renal failure	73 (16.4)
Osteoarthritis	249 (55.9)
Cardiovascular disorders	119 (26.8)
Diabetes	64 (14.4)
Liver disorders	36 (8.1)
Neoplasms	28 (6.29)
Sangha comorbidity index (0 to 36)	4 (2 to 7)
Fulfils preliminary ACR criteria	411/445 (92.4)
Disease duration (years)	3.8 (1.5 to 10.1)
Joint involvement	
Monoarticular (1 joint)	95(21.5)
Oligoarticular (2 to 4 joints)	264 (59.7)
Polyarticular (>4 joints)	83 (18.8)
Swollen joints (0 to 66)	0 (0 to 1)
Tender joints (0 to 68)	1 (0 to 3)
Presence of tophi^a^	87 (19.9)
Number of flares (12 months)	1 (0 to 3)
Flare (previous month)	132 (29.9)
Serum urate level (mg/dl)^b^	6.3 (1.7)
Previous corticosteroids	125 (28.0)
Current NSAIDs or colchicine	189 (42.4)
Allopurinol	303 (67.9)
Febuxostat	60 (13.4)

Data presented as *n *(%), mean (standard deviation) or median (interquartile range). ACR, American College of Rheumatology; NSAID, nonsteroidal anti-inflammatory drug. Data available for ^a^437 subjects and ^b^410 subjects.

The study sample included a majority of male (90%) patients, with an overall mean (standard deviation) age of 63.9 (11.6) years, and a high frequency of Caucasian subjects (99%). Male patients were younger than female patients with a mean (standard deviation) age of 63.6 (11.6) and 66.9 (11.8) years, respectively. The education background of the patients was mainly secondary school or lower. The majority of patients were overweight or obese and showed a high prevalence of high alcohol intake. More than 90% of patients had at least one comorbid condition according to the Self-Administered Comorbidity Questionnaire, mostly of a metabolic or cardiovascular nature. Most subjects had more than one comorbid condition with a median number (interquartile range) of 3 (1 to 4). More than 92% of patients fulfilled the preliminary American College of Rheumatology criteria for disease classification (22% only based on crystal identification), and showed a median of 3.8 years of disease duration and a high prevalence of oligoarticular or polyarticular disease. About 30% of patients experienced an attack of gout within 1 month prior to the clinical evaluation, and 214 out of 410 (52.2%) had sUA levels above 6 mg/dl, despite 361/446 (80.9%) being on ULT.

**Figure 1:**
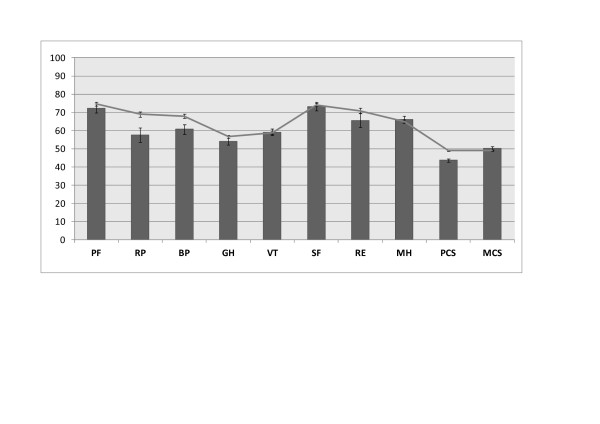
**Health-related quality of life in gout patients compared with that expected for Italian normative population**. Bar chart: Short Form-36 subscales and summary scales - physical functioning (PF), role physical (RP), bodily pain (BP), general health (GH), vitality (VT), social functioning (SF), role emotional (RE), mental health (MH), Physical Component Summary (PCS) and Mental Component Summary (MCS) scale scores. Line chart: normative data for Italian population. Error bars represent 95% confidence intervals (CIs). Nonoverlapping CIs indicate statistically significant impairment.

According to the HAQ-DI score, at least moderate functional disability was observed in 94 out of 444 (21.1%) patients, of whom 72 (16.22%) showed moderate disability and 22 (4.95%) showed severe disability (Figure 1). The median (interquartile range) HAQ score was 0.25 (0 to 0.875). Overall, the physical components summarised by the PCS score were reduced as compared with the expected score for the age-matched and gender-matched general population (Figure 1). The MCS score did not differ from that for the general population.

Associations of sociodemographic, general health and disease-specific variables with functional disability and HRQoL were evaluated. The results of crude and adjusted analyses are presented in Tables 2 and 3.

**Table 2:** Association between sociodemographic, general health and disease-specific variables to functional disability^a^

Variable	HAQ-DI		
	
	Crude OR (95% CI)	Adjusted OR (95% CI)^b^	Adjusted OR (95% CI)^c^
**Sociodemographic **			
Gender (female vs. male)	6.27 (3.37, 11.64)		
Age (5 years)	1.28 (1.15, 1.44)		
Education			
No or primary	Reference		
Secondary	0.48 (0.27, 0.84)	0.66 (0.36, 1.20)	
Upper secondary	0.21 (0.10, 0.42)	0.27 (0.13, 0.56)	
Tertiary	0.17 (0.05, 0.57)	0.19 (0.05, 0.73)	
Employment			
Employed	Reference		
Unemployed	5.85 (2.21, 15.48)	2.49 (0.85, 7.31)	
Retired	3.57 (1.81, 7.03)	2.21 (0.92, 5.28)	
Unknown	-	-	
**General health variables **			
Comorbidity index	1.22 (1.15, 1.29)	1.16 (1.09, 1.24)	
Body mass index			
Normal	Reference		
Overweight	1.82 (0.90, 3.68)	2.44 (1.14, 5.22)	
Obese	2.71 (1.28, 5.73)	4.02 (1.78, 9.07)	
Current smokers	0.99 (0.53, 1.85)	1.37 (0.71, 2.64)	
High alcohol consumers	0.96 (0.59, 1.54)	1.46 (0.87, 2.45)	
**Gout-related variables**			
Disease duration (>5 years)	1.98 (1.24, 3.14)	2.12 (1.30, 3.46)	2.03 (1.22, 3.40)
Joint involvement			
Monoarticular	Reference		
Oligoarticular (2 to 4 joints)	1.75 (0.89, 3.45)	1.99 (0.96, 4.09)	1.49 (0.70, 3.16)
Polyarticular (>4 joints)	3.67 (1.72, 7.82)	4.88 (2.17, 10.97)	3.82 (1.63, 8.95)
Number of attacks last year	1.10 (1.05, 1.17)	1.13 (1.06, 1.20)	1.11 (1.04, 1.18)
Attacks last month	1.89 (1.17, 3.04)	2.33 (1.40, 3.89)	2.20 (1.27, 3.81)
Presence of tophi	2.14 (1.28, 3.59)	2.06 (1.19, 3.56)	1.92 (1.07, 3.43)
Number of swollen joints	1.25 (1.16, 1.35)	1.25 (1.15, 1.35)	1.23 (1.13, 1.33)
Number of tender joints	1.14 (1.10, 1.19)	1.13 (1.08, 1.18)	1.10 (1.06, 1.15)
Serum uric acid			
<5 mg/dl	Reference		
5 to 6 mg/dl	1.18 (0.57, 2.42)	1.25 (0.58, 2.69)	1.24 (0.56, 2.74)
6 to 7 mg/dl	0.94 (0.42, 2.10)	1.20 (0.51, 2.79)	0.83 (0.34, 2.06)
>7 mg/dl	1.72 (0.89, 3.32)	2.49 (1.22, 5.06)	1.81 (0.87, 3.74)
Urate-lowering treatment	1.02 (0.57, 1.81)	1.11 (0.59, 2.09)	1.06 (0.55, 2.03)
Current NSAIDs or colchicine	3.17 (1.89, 5.33)	3.24 (1.87, 5.61)	2.57 (1.40, 4.72)
Previous corticosteroids	1.87 (1.15, 3.05)	1.76 (1.04, 2.97)	1.64 (0.93, 2.89)

NSAID, nonsteroidal anti-inflammatory drug. ^a^Assessed by the Health Assessment Questionnaire Disability Index (HAQ-DI). ^b^Adjusted for age and gender. ^c^Adjusted for age, gender, comorbidities, body mass index, smoking, high alcohol consumption, education and employment (on 10 imputed datasets).

**Table 3:** Association between sociodemographic, general health and disease-specific variables to health-related quality of life^a^

	SF-36 PCS			SF-36 MCS		
	
	Crude MD (95% CI)	Adjusted MD (95% CI)^b^	Adjusted MD (95% CI)^c^	Crude MD (95% CI)	Adjusted MD (95% CI)^b^	Adjusted MD (95% CI)^c^
**Sociodemographic variables**						
Gender (female vs. male)	-7.37 (-10.67, -4.08)			-3.45 (-6.44, -0.45)		
Age (5 years)	-1.04 (-1.45, -0.64)			-0.15 (-0.52, 0.23)		
Education						
No or primary	Reference			Reference		
Secondary	1.44 (-0.96, 3.84)	-0.26 (-2.69, 2.18)		0.52 (-1.68, 2.73)	0.42 (-1.90, 2.73)	
Upper secondary	5.71 (3.32, 8.10)	3.95 (1.48, 6.41)		2.15 (-0.06, 4.35)	2.11 (-0.24, 4.45)	
Tertiary	5.40 (1.84, 8.97)	3.84 (0.26, 7.41)		2.30 (-0.98, 5.58)	2.46 (-0.94, 5.86)	
Employment						
Employed	Reference			Reference		
Unemployed	-6.05 (-10.16, -1.94)	-3.10 (-7.31, -1.11)		-2.74 (-6.50, 1.03)	-1.83 (-5.77, 2.12)	
Retired	-4.59 (-6.69, -2.49)	-1.99 (-4.85, 0.88)		-0.21 (-2.13, 1.71)	0.34 (-2.34, 3.01)	
Unknown	-	-		-	-	
**General health variables **						
Comorbidity index	-1.12 (-1.34, -0.90)	-0.98 (-1.22, -0.74)		-0.58 (-0.80, -0.37)	-0.62 (-0.85, -0.39)	
Body mass index						
Normal	Reference			Reference		
Overweight	-2.4 7 (-4.97, 0.04)	-2.98 (-5.38, -0.58)		-1.06 (-3.32, 1.20)	-1.45 (-3.71, 0.81)	
Obese	-4.87 (-7.73, -2.01)	-5.79 (-8.51, -3.06)		-2.35 (-4.92, 0.22)	-2.72 (-5.29, -0.16)	
Current smokers	0.04 (-2.60, 2.67)	-0.90 (-3.45, 1.64)		0.47 (-1.9, 2.84)	0.3 (-2.08, 2.68)	
High alcohol consumers	0.57 (-1.47, 2.60)	-0.58 (-2.56, 1.39)		-0.94 (-2.77, 0.88)	-1.33 (-3.17, 0.52)	
**Gout-related variables**						
Disease duration (>5 years)	-2.54 (-4.49, -0.58)	-2.37 (-4.25, -0.49)	-1.70 (-3.45, 0.05)	-2.54 (-4.29, -0.79)	-2.61 (-4.35, -0.86)	-2.08 (-3.79, -0.37)
Joint involvement						
Monoarticular	Reference			Reference		
Oligoarticular (2 to 4 joints)	-3.13 (-5.53, -0.73)	-3.35 (-5.64, -1.06)	-2.22 (-4.38, -0.07)	0.40 (-1.78, 2.57)	0.27 (-1.90, 2.44)	0.96 (-1.18, 3.10)
Polyarticular (>4 joints)	-5.66 (-8.69, -2.62)	-5.92 (-8.82, -3.01)	-3.79 (-6.55, -1.04)	-1.05 (-3.80, 1.70)	-1.25 (-4.00, 1.50)	0.42(-2.30, 3.16)
Number of attacks last year	-0.70 (-0.94, -0.47)	-0.71 (-0.93, -0.49)	-0.56 (-0.77, -0.36)	-0.45 (-0.66, -0.24)	-0.44 (-0.65, -0.23)	-0.34 (-0.55, -0.13)
Attacks last month	-8.18 (-10.15, -6.20)	-8.62 (-10.49, -6.76)	-8.13 (-9.88, -6.38)	-3.22 (-5.09, -1.35)	-3.33 (-5.20, -1.47)	-2.72 (-4.58, -0.86)
Presence of tophi	-4.72 (-7.15, -2.30)	-4.16 (-6.50, -1.83)	-3.20 (-5.41, -0.99)	0.56 (-1.62, 2.75)	0.71 (-1.47, 2.89)	1.26 (-0.88, 3.40)
Number of swollen joints	-0.91 (-1.17, -0.65)	-0.78 (-1.04, -0.53)	-0.54 (-0.79, -0.29)	-0.40 (-0.64, -0.16)	-0.37 (-0.62, -0.13)	-0.20 (-0.45, 0.04)
Number of tender joints	-0.64 (-0.79, -0.49)	-0.58 (-0.73, -0.42)	-0.39 (-0.55, -0.24)	-0.38 (-0.52, -0.24)	-0.36 (-0.51, -0.21)	-0.24 (-0.39, -0.09)
Serum uric acid						
<5 mg/dl	Reference			Reference		
5 to 6 mg/dl	-0.09 (-3.14, 2.96)	-0.15 (-3.06, 2.76)	-0.60 (-3.24, 2.02)	-0.2 (-2.97, 2.57)	-0.23 (-2.99, 2.52)	-0.66 (3.19, 1.86)
6 to 7 mg/dl	-0.12 (-3.09, 2.86)	-0.60 (-3.46, 2.26)	0.48 (-2.32, 3.29)	-1.68 (-4.38, 1.03)	-1.71 (-4.42, 0.99)	-0.75 (-3.55, 2.04)
>7 mg/dl	-1.78 (-4.56, 0.99)	-2.95 (-5.63, -0.28)	-2.06 (-4.51, 0.38)	-1.45 (-3.98, 1.07)	-1.78 (-4.31, 0.76)	-1.00 (-3.39, 1.38)
Urate-lowering treatment	0.41 (-2.05, 2.87)	0.37 (-2.01, 2.75)	0.49 (-1.71, 2.69)	0.27 (-1.94, 2.47)	0.06 (-2.16, 2.27)	0.03 (-2.12, 2.19)
Current NSAIDs or colchicine	-7.03 (-8.95, -5.11)	-6.67 (-8.52, -4.82)	-5.47 (-7.38, -3.56)	-2.79 (-4.57, -1.00)	-2.53 (-4.32, -0.75)	-2.13 (-4.04, -0.23)
Previous corticosteroids	-5.10 (-7.26, -2.95)	-4-67 (-6.75, -2.59)	-3.51 (-5.46, -1.26	-4.15 (-6.06, -2.23)	-3.90 (-5.83, -1.98)	-3.39 (-5.30, -1.48)

^a^Assessed by the Short Form-36 (SF-36) questionnaire, physical and mental summary score. CI, confidence interval; MCS, Mental Component Summary; MD, mean difference; PCS, Physical Component Summary. ^a^Adjusted for age and gender. ^b^Adjusted for age, gender, comorbidities, body mass index, smoking, high alcohol consumption, education and employment (on 10 imputed datasets).

Among sociodemographic data, older age, female gender and lower education significantly affected functional disability and the physical component of the SF-36 while only female gender influenced the mental component.

General health variables that significantly associated with higher HAQ-DI and lower PCS scores in age-adjusted and gender-adjusted models included comorbidities, overweight and obesity. Obesity was also associated with a statistically and clinically relevant lower MCS score.

Disease-related variables were strongly associated with functional disability and the physical component in the unadjusted analyses. After adjusting for age, gender and the other confounders, variables indicating chronic disease (disease duration, polyarticular joint involvement, presence of tophi) and uncontrolled joint inflammation (swollen and tender joints, attacks in the last months and current use of nonsteroidal anti-inflammatory drugs or colchicine) still showed statistically and clinically significant associations with the HAQ-DI and PCS scores. The MCS score was only influenced by acute symptom-related variables.

Neither uncontrolled sUA nor ULT were associated with HAQ-DI or PCS outcomes in the fully adjusted analyses, even though subjects with high sUA levels (>7 mg/dl) showed statistically and clinically meaningful worse outcomes the analyses adjusted for age and gender.

**Figure 2:**
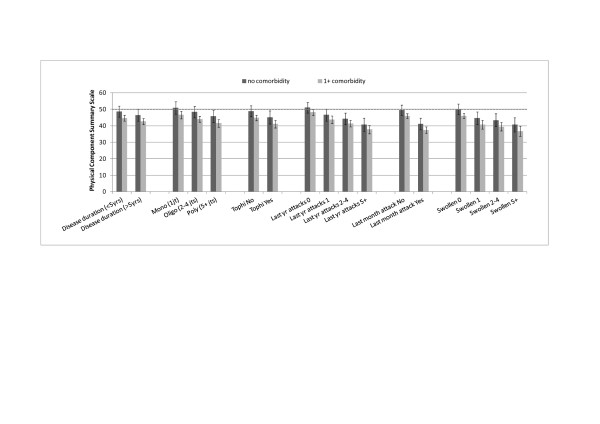
**Mean Physical Component Summary scores according to different disease characteristics and presence of comorbidities**. Error bars represent 95% confidence intervals (CIs). CIs not crossing the expected value of 50 (dashed line) indicate statistically significant impairment.

Adjusted mean PCS scores, stratified by category of disease severity and presence of comorbidity, showed that subjects with monoarticular disease, absence of tophi and intercritical disease did not show significant impairment in the physical component of the PCS (Figure 2).

## Discussion

Our results show that functional and HRQoL impairment observed in gout patients is not only due to general health variables, such as associated risk factors and comorbidities, but is also influenced by the activity and severity of several disease-related variables. Two studies previously attempted to answer the same question as the current study at a population level, suggesting that gout is not independently associated with HRQoL impairment. The first, a case-control study carried out in the United Kingdom using electronic records of general practitioners, reported an association between gout and impaired overall quality of life in a univariable analysis. Association was only evident in the physical domain of the World Health Organization Quality of Life - BREF instrument after adjusting for age, gender, body mass index, and medical/musculoskeletal comorbidities [12]. This association was only slightly statistically significant and of unclear clinical relevance. A second population-based survey, carried out from the US veterans database, found poorer HRQoL and higher functional limitation in patients with gout in unadjusted analyses that disappeared after controlling sociodemographic and comorbidity data [13]. These population studies, even limited by low response rates and incomplete clinical assessment, certainly captured the real distribution of gout severity, including a higher prevalence of cases with mild disease.

In our study we included a probability sample of patients with a clinical diagnosis of gout confirmed by a rheumatologist and we assessed functional disability and HRQoL using the tools recommended by OMERACT [10]. All patients were also directly assessed for sociodemographic, lifestyle and clinical variables in order to control a number of potential confounders. The results of the fully-adjusted models indicated that the association between disease-specific variables and functional disability, as assessed by HAQ-DI and PCS, are both statistically and clinically meaningful. To estimate the specific influence of disease-related variables on the PCS compared with the expected from the general population, the multivariable models were applied to obtain the adjusted mean PCS score for different levels of disease severity variables. The subgroup of patients with monoarticular disease, a low number of attacks and an absence of tophi did not show any significant impairment of the physical component of the SF-36 when compared with that of the general population. This result may partially explain the weakness of independent association between gout and disability or poor HRQoL in the general population given that population-based studies are likely to have a considerable number of subjects with a single attack or intercritical gout [12,13]. Disease-associated impairment of HRQoL thus seems to be related to a subgroup of patients with chronic gout while the physical impairment of subjects with mild disease is mainly due to general risk factors and concurrent medical conditions.

In our study, another significant finding is the identification of a number of disease-related factors associated with a worse outcome. The number of joints cumulatively involved was one of the major factors independently associated with increased presence of functional disability and worsening of the SF-36 physical component. This has been previously demonstrated for the number of involved joints against the PCS and MCS and for the disease-specific HRQoL instrument Gout Impact Scale [8,17]. Other findings are also highly consistent with the existing literature: the presence of tophi, as well as factors indicating active disease (such as number of swollen and tender joints, frequency of attacks) [16,17,34], have been reported as major predictors of functional disability [14,16] and poor HRQoL [16,34]. Our data confirm such associations and provide fully adjusted estimates by analysing the impact of every single variable using clinically relevant cutoff values or meaningful differences from tools validated for gout.

Our study confirms the lack of association between cross-sectional sUA levels and HAQ-DI or SF-36 dimensions [12,14,16], while other studies reported a significant association with longitudinal levels of sUA [9,34]. This result might be partially explained by the fact that patients with more severe disease might be treated more aggressively, and therefore they exhibit a relatively better level of sUA than patients with mild disease. Furthermore, despite the relevance of sUA as a biomarker in gout [11], the effect on the outcome of severe disease is likely to be reached by a persistent control of sUA over time [35].

The mental component of the SF-36 is not generally affected in patients with gout [12,13,17,35]. Main clinical characteristics influencing the MCS score are variables of acute disease such as gout attacks, tender joints and the daily use of nonsteroidal anti-inflammatory drugs [12,16]. Our results confirmed these previous findings and further showed that the use of systemic glucocorticoids may also impact on the mental component of the SF-36. This finding, already reported by Roddy and colleagues [12], should be interpreted in the context of more severe disease leading to the prescription of corticosteroids rather than as an effect on disease severity - an issue that is still to be resolved.

This study has some limitations. Results may not be translated to the whole population of gout patients since participants were recruited from the registries of rheumatology clinics that usually follow up patients with chronic disease and atypical presentations. However, demographic and clinical characteristics are consistent with those reported in a recent descriptive study carried out in a nationwide representative primary care register in Italy [2]. Given that the majority of rheumatology clinics were hospital based, recruitment of patients can be further biased towards gout patients with more severe comorbidities. However, a detailed clinical evaluation in a rheumatological setting has included patients with accurate diagnosis of gout (92% fulfilled the preliminary American College of Rheumatology criteria) and allowed analyses for different levels of disease severity. The cross-sectional design is essentially descriptive and exploratory since it does not allow for the ascertainment of temporal relationship between clinical characteristics, treatment or modification of risk factors on outcomes.

## Conclusions

The KING study provides evidence for an independent association of gout and gout-related characteristics with functional outcome and HRQoL. In patients with chronic gout, the study also confirms the well-known influence of sociodemographic characteristics and comorbidities on the disease outcomes. These findings support the need for an integrated therapeutic approach, which combines specific disease control to limit progression of acute or intercritical to chronic gout and management of concomitant risk factors and medical conditions.

## Abbreviations

HAQ-DI: Health Assessment Questionnaire Disability Index; HRQoL: health-related quality of life; KING: Kick-off of the Italian Network for Gout; MCS: Mental Component Summary; OMERACT: Outcome Measures in Rheumatology Clinical Trial; PCS: Physical Component Summary; SF-36: Short Form-36; SIR: Italian Society for Rheumatology; sUA: serum uric acid; ULT: urate-lowering treatment; VT: vitality.

## Competing interests

The authors declare that they have no competing interests.

## Authors' contributions

CAS, MAC, MM, CM, MG, GM, FS, LP and MM-C planned the study. All the authors collected data. CAS, MCM and GC analysed data. CAS supervised the project and wrote the first draft. All authors critically revised and approved the final manuscript.

## Supplementary Material

Additional file 1 **is a flowchart of the KING study (cross-sectional)**. Click here for file
