# Supplementary figures and images for: Gout impacts on function and health-related quality of life beyond associated risk factors and medical conditions: results from the KING observational study of the Italian Society for Rheumatology (SIR)
Source: Arthritis Res Ther. 2013 Sep 3;15(5):R101. doi: 10.1186/ar4281 (PMC3979095; doi:10.1186/ar4281)

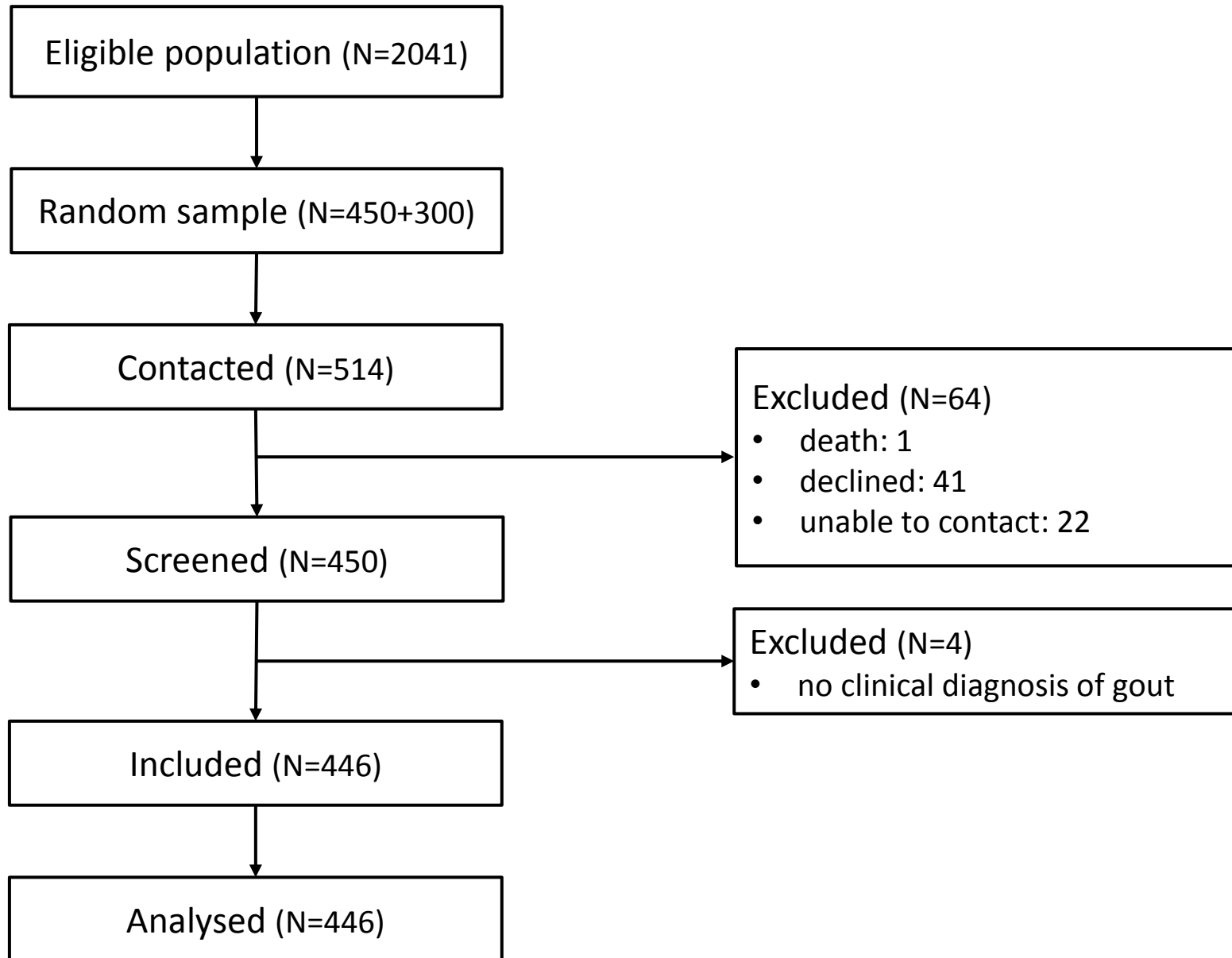

Supplement: Additional file 1 — is a flowchart of the KING study (cross-sectional). [file ar4281-S1.PDF]
